# Effects of Different Growth Media on In Vitro Seedling Development of an Endangered Orchid Species *Sedirea japonica*

**DOI:** 10.3390/plants10061193

**Published:** 2021-06-11

**Authors:** Jiae An, Pyoung Beom Kim, Hyeong Bin Park, Seongjun Kim, Hwan Joon Park, Chang Woo Lee, Byoung-Doo Lee, Nam Young Kim, Jung Eun Hwang

**Affiliations:** 1 Division of Restoration Research, Research Center for Endangered Species, National Institute of Ecology, Yeongyang 36531, Korea; jiae_an@nie.re.kr (J.A.); kimpb89@nie.re.kr (P.B.K.); phb1274@nie.re.kr (H.B.P.); dao1229@nie.re.kr (S.K.); rhg9281@nie.re.kr (H.J.P.); jacky903@nie.re.kr (C.W.L.); bdlee@nie.re.kr (B.-D.L.); skadud2@nie.re.kr (N.Y.K.); 2 Department of Environmental Science and Ecological Engineering, Korea University, Seoul 02841, Korea; 3 Department of Life Science, Yeungnam University, Gyeongsan 38541, Korea

**Keywords:** Hyponex media, Murashige and Skoog media, organic supplementation, *Sedirea japonica*, seedling growth, tissue culture

## Abstract

*Sedirea japonica* is becoming endangered, and even extinct, due to habitat destruction and illegal collection, and the development of an optimized artificial propagation system is necessary for its conservation and reintroduction. Thus, the effects of plant growth medium strength (Murashige and Skoog (MS) and Hyponex media) and the addition of activated charcoal (AC) and organic supplements on seedling growth of *S. japonica* were investigated through in vitro seed culture. The results showed that seedling growth was higher in half-strength (1/2) media than in full-strength media. After the addition of AC, the highest leaf area (2.14 cm^2^) was recorded in the seedlings grown in 1/2 Hyponex medium, and after the addition of organic supplements, root development increased regardless of the media type. Among the sixteen suitable media tested at later seedling growth stages, 1/2 MS medium with the addition of 0.6 g·L^−1^ AC, 30 g·L^−1^ banana homogenate and 10 g·L^−1^ apple homogenate was generally effective in fresh weight (6.13 g) and root length (9.59 cm). We demonstrated which organic supplements are preferred for in vitro growth of seedlings developed from *S. japonica* protocorms by asymbiotic seed culture, which can be used for mass production and conservation of this rare epiphytic orchid.

## 1. Introduction

Biodiversity conservation is one of the main issues worldwide because the survival of each species is necessary for the healthy functioning of an ecosystem. Flowering plants are divided into five subfamilies, and the Orchidaceae is one of the largest and most widespread families which has about 28,000 currently accepted species, accounting for about 8% of angiosperm species diversity [1,2]. However, the International Union for Conservation of Nature (IUCN) listed 948 orchid species on the IUCN Global Red list in 2017, 56.5% of which are threatened [3]. Orchids are one of the most important global cut flowers and pot plants [4]. They require optimum conditions to survive in an ecosystem because they are the most evolved of all flowering plants [5]. In addition, orchids are a good indicator of a healthy, functioning ecosystem because they interact with other plants, fungi, and animals for their germination and specific pollinations (IUCN website). Despite the importance of orchids, approximately a quarter of all orchid species are endangered because of habitat destruction, plant smuggling, global climate change, and overexploitation [6,7]. Multifaceted integrative approaches are required to facilitate the ex situ and in situ conservation of orchid species. Thus, the use of in vitro tissue culture seems to be a promising approach and advisable to save the species from extinction.

In vitro tissue culture is a viable alternative technique for rapid mass multiplication, and it is widely used for the conservation of endangered orchids. There are several reports on establishing optimal culture conditions for each orchid species such as *Calanthe tricarinata* [6], *Cyrtopodium punctatum* [8], *Pecteilis Radiata* [9], and *Gastrochilus Calceolaris* [10]. To estimate the culture protocols of orchid seeds in vitro, it is important to consider several factors such as pod maturity, components of culture media, and light and temperature conditions [11]. The composition of culture media is a significant factor in seedling growth. Carbohydrates, amino acids, vitamins, mineral nutrients, growth hormones, and organic acids are necessary for in vitro asymbiotic embryo development and protocorm formation [12]. Activated charcoal (AC), plant growth regulators, and organic growth supplements have also been shown to influence in vitro regeneration, multiplication, and growth of orchid seedlings [9,10]. As these components exhibit a strong species-specific impact, their content in the medium must be determined and provided according to the distinct requirements of a given orchid species [11,13]. In *Hadrolaelia grandi,* culture medium with activated charcoal acted as an important factor in plant development [14]. Kim et al. [9] investigated the effects of activated charcoal and culture medium strength on seed germination and seedling development of *Pecteilis radiate*. The effects of organic growth additives have been tested in a large number of orchids, such as *Cypripedium formosanum* [15], *Phalaenopsis* [16], *Dendrobium* *species* [17], *Zygopetalum mackayi* [18], and *Cymbidium pendulum* [19]. Among these factors, we would like to determine which medium composition factors have the most important influence on the growth of *Sedirea japonica*.

*Sedirea japonica* is an economically important orchid species indigenous to the subtropical regions of southern China, Japan, and Korea [20]. In Korea, this species, known as ‘Nadopungran,’ is an evergreen epiphyte that attaches to trees or rocks and is distributed in the Bijarim Forest on Jeju island at 150 m above sea level, the pine forests on Jollanamdo, and the southern islands. The roots, leaves, and flowers of *S. japonica* all have ornamental value. The flowers are light green with red patterns on the lips, the flower size is 5−15 cm, and 4−10 flowers grow on each flower stalk. Its scent is deep, making it a popular species for horticulture [21]. Much previous research has studied the molecular, biological, and physiological properties of *S. japonica* flowers to fully understand their properties. For instance, Jiang et al. [20] isolated the pistillata-like (PI-like) gene involved in flower morphogenesis in model plants. This gene has an important physiological function in fluorescence. In addition, Bean et al. [22] and Kim et al. [23] studied the expression pattern of the fragrance of flowers. However, it is thought that after years of excessive and reckless harvesting for horticulture, wild *S. japonica* that once grew in Korea has become extinct. Therefore, this orchid has been designated as a critically endangered species (CR) and is regionally protected by law [24]. Thus, it is strongly recommended to multiply the species, possibly through tissue culture techniques, to save its wild populations from extinction. Previous studies reported the effect of polyvinyl pyrrolidone (PVP), activated charcoal (AC), and mineral composition of medium on in vitro seed germination of *S. japonica* [25]. Although there are reports of the effect of growth retardants on *S. japonica* [26,27], none of them examine in great depth the influence of culture media and incubation conditions on the speed of seedling development. Therefore, this study aims to initiate in vitro cultures through the asymbiotic seed germination technique and test the efficacy of plant growth regulators and organic growth supplements on the speed of seedling development in *S. japonica*.

In the present study, we evaluated the effects of different strengths of Murashige and Skoog (MS) and Hyponex (Kisan Bio, Seoul, Korea) media and the effects of supplementation with charcoal, banana homogenate and apple homogenate on the growth and development of *S. japonica*. We established a reproducible protocol for the rapid growth of *S. japonica* seedlings by in vitro culture. The results of this report will be helpful for conservation practitioners to save time in developing plantlets that can be used for the reintroduction of this species to the wild.

## 2. Results

### 2.1. Effects of Different Strengths of MS and Hyponex Media on Seedling Growth

**Figure 1:**
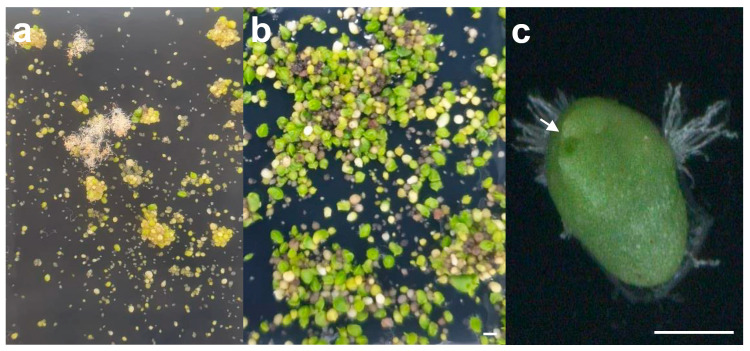
*Sedirea japonica* seed germination and protocorm development. (**a**) Seed embryos were swollen and seed coats were ruptured one month after sowing; (**b**,**c**) Shoot apices (indicated by arrows) were formed from embryos two months after sowing. Scale bar = 1 mm.

In the present study, seeds from the same pod were transferred to the Hyponex medium, and protocorm and shoot apex developed one month (Figure 1a) and two months (Figure 1b,c) after sowing, respectively. Embryos with shoot apices formed were transferred to each culture bottle of different medium types, strengths, and compositions.

**Figure 2:**
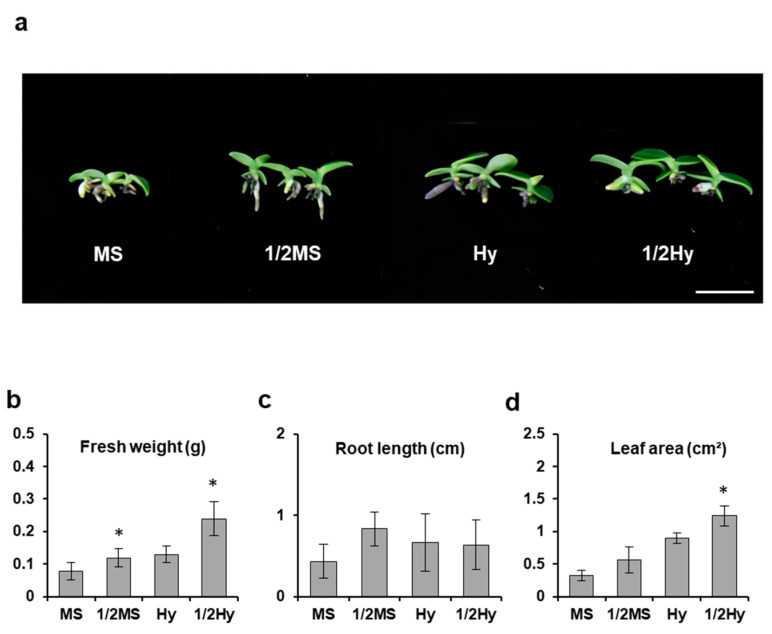
Effects of culture media strength on *Sedirea japonica* seedling growth. (**a**) Growth differences in seedlings were determined seven months after sowing. The pictures show a representative seedling of each medium. Fresh weight (**b**), root length (**c**), and leaf area (**d**) were measured. Scale bar = 2 cm. MS: full-strength MS; 1/2MS: half-strength MS; Hy: full-strength Hyponex; 1/2Hy: half-strength Hyponex. Data are shown as mean ± SD of the values obtained from triplicate experiments. * *p* ≤ 0.05.

Our results showed that the type and concentration of the basal medium influenced the response of seedlings in terms of growth. All tested media supported protocorm growth (Figure 2). Early seedling growth was investigated by comparing fresh weight, root length, and leaf area of the plants seven months after sowing. Advanced seedling development was significantly affected by the composition of the medium when measured by seedling fresh weight rate (Figure 2b). In our result, seedlings grown in half-strength MS media (1/2 MS) had higher fresh weights (0.118 g) than the weights of seedlings grown in full-strength MS media (MS) (0.007 g). The fresh weight of seedlings grown in half-strength Hyponex media (1/2 Hyponex) was 0.239 g, which was 1.8-fold higher than that of seedlings grown in full-strength Hyponex media (Hyponex) (0.129 g). Regarding the effect of different media strength on leaf area, seedlings grown in 1/2 Hyponex media had the greatest leaf area (1.238 cm^2^), while there was no significant effect on the root length.

### 2.2. Effects of Supplementation with Activated Charcoal on Seedling Growth

**Figure 3:**
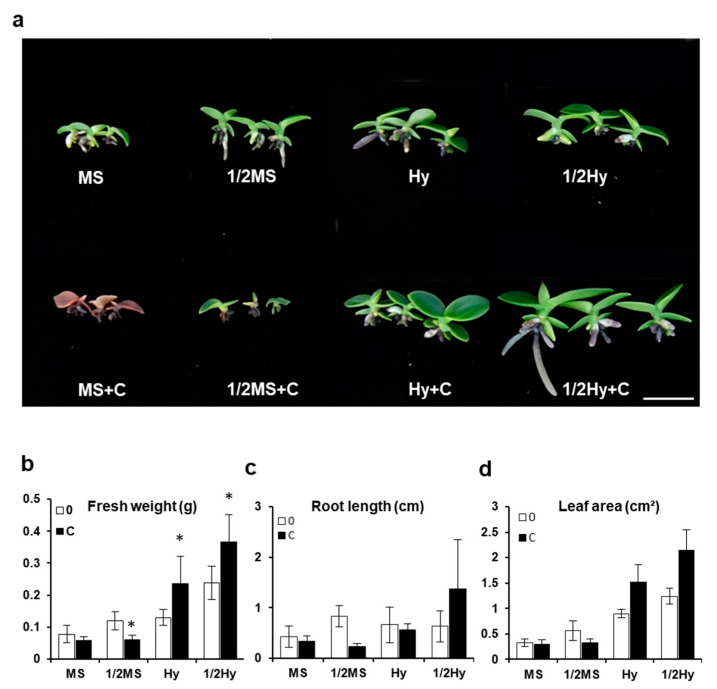
Effect of activated charcoal (AC) on *Sedirea japonica* seedling growth. (**a**) Growth differences in seedlings were determined seven months after sowing. The pictures show a representative seedling of each medium. Fresh weight (**b**), root length (**c**), and leaf area (**d**) were measured. Scale bar = 2 cm. C: activated charcoal; MS: full-strength MS; 1/2MS: half-strength MS; Hy: full-strength Hyponex; 1/2Hy: half-strength Hyponex. Data are shown as mean ± SD of the values obtained from triplicate experiments. * *p* ≤ 0.05.

An experiment was conducted to compare the effects of activated charcoal (AC) on early seedling growth seven months after sowing, and the results are shown in Figure 3. Firstly, detrimental effects were observed in seedlings in the MS and 1/2 MS media supplemented with AC: most seedlings in cultures in the MS medium showed necrosis (browning) of their basal part, and in cultures in the 1/2 MS medium, the basal part of the seedlings was browning or green with few or no roots (Figure 3a). Moreover, when seedlings were grown in Hyponex and 1/2 Hyponex media, their fresh weight was higher in media with AC (0.236 g and 0.366 g, respectively; Figure 3b) than in media without AC (0.129 g and 0.239 g, respectively). The root length of seedlings was not significantly different between Hyponex and 1/2 Hyponex media with and without AC (Figure 3c). The leaf area of seedlings increased approximately twofold when AC was added to Hyponex and 1/2 Hyponex media compared to that without AC (Figure 3d). Additionally, seedlings grown in MS and 1/2 MS media supplemented with AC had lower fresh weight, root length, and leaf area than those of seedlings grown in MS and 1/2 MS media without AC (Figure 3b–d).

### 2.3. Effects of Supplementation with Banana and Apple Homogenate on Seedling Growth

**Figure 4:**
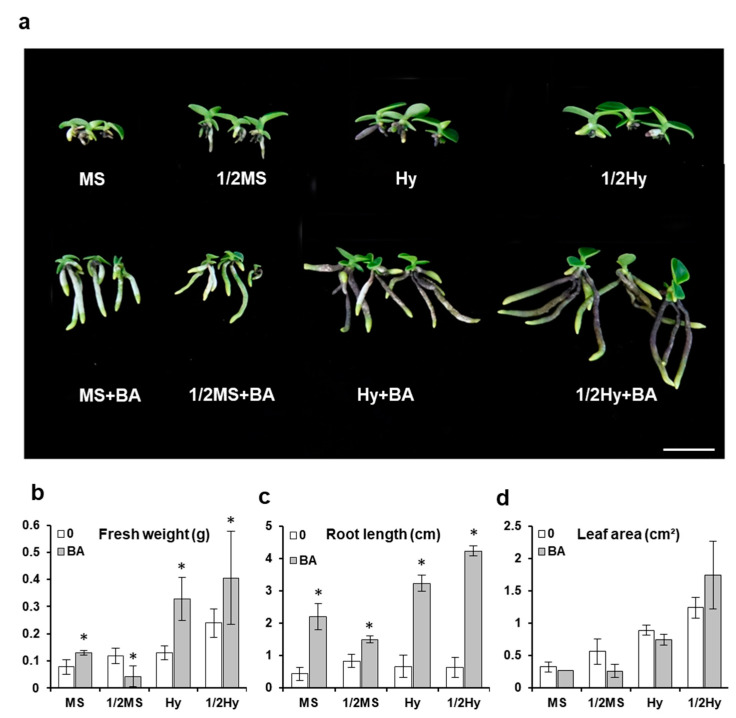
Effect of banana and apple homogenate (BA) on *Sedirea japonica* seedling growth. (**a**) Growth differences in seedlings were determined seven months after sowing. The pictures show a representative seedling of each medium. Fresh weight (**b**), root length (**c**), and leaf area (**d**) were measured. Scale bar = 2 cm. MS: full-strength MS; 1/2MS: half-strength MS; Hy: full-strength Hyponex; 1/2Hy: half-strength Hyponex. Data are shown as mean ± SD of the values obtained from triplicate experiments. * *p* ≤ 0.05.

The effects of organic supplementation with banana homogenate (BH) and apple homogenate (AH) on seedling growth of *S. japonica* were studied. We found that the organic supplementation of BH and AH had significant effects on the growth and development of *S. japonica* in terms of root elongation, leaf size, and plantlet elongation on early seedling growth (Figure 4). Our results indicated that among the different media conditions studied, organic supplementation was the most effective in promoting root growth. The root length of the seedlings increased twofold and fourfold after the addition of banana and apple homogenate (BA) (Figure 4c). The root lengths of seedlings cultured in different media containing BA were 2.2 cm (MS), 1.5 cm (1/2 MS), 3.2 cm (Hyponex), and 4.2 cm (1/2 Hyponex). Similarly, the fresh weights and leaf areas of seedlings increased slightly when grown with BA compared to those grown without BA (Figure 4b,d).

### 2.4. Effects of Different Media on Plant Growth at Later Plant Development Stages

**Figure 5:**
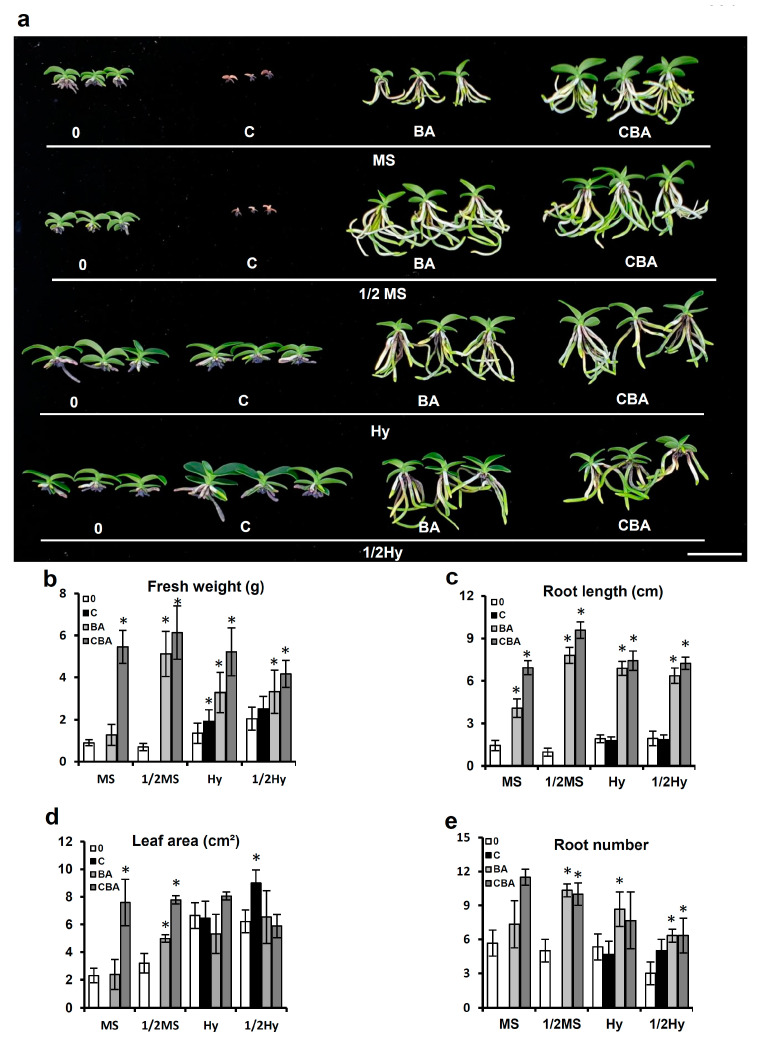
Effect of different medium on plant growth at the later stage of *Sedirea japonica*. (**a**) Growth differences in seedlings were determined nine months after sowing. The pictures show a representative seedling of each medium. Fresh weight (**b**), root length (**c**), leaf area (**d**), and root number (**e**) were measured. Scale bar = 2 cm. C: activated charcoal; BA: banana and apple homogenates; MS: full-strength MS; 1/2MS: half-strength MS; Hy: full-strength Hyponex; 1/2Hy: half-strength Hyponex. Data are shown as mean ± SD of the values obtained from triplicate experiments. * *p* ≤ 0.05.

An experiment was carried out to compare the effects of different media on seedling growth at later plant development stages, 9 months after sowing. Figure 5 shows the different effects of AC and BA supplements on seedling growth at late plant development stages in MS and Hyponex media. We found that organic supplementation of BA had significant effects on root and shoot development (Figure 5). In full- and half-strength MS and Hyponex media with BA, fresh weight and root length were increased significantly compared to those in these media without BA. However, the addition of AC did not significantly influence these parameters. These results indicated that organic supplementation had a greater effect on the development of *S. japonica* than AC supplementation.

The fresh weights and root lengths of plants grown in MS and 1/2 MS media with AC and BA were approximately six times higher than those of plants grown in MS and 1/2 MS media without AC and BA (Figure 5b,c). Maximum rooting was observed in the MS medium supplemented with AC and BA together, and the number of roots increased approximately two times in MS and 1/2 MS media with these supplements, respectively (Figure 5e). Root and shoot development was also promoted in the Hyponex medium by BA or AC and BA additives, but there was little change compared to those in the MS medium.

## 3. Discussion

In vitro seedling growth of orchids greatly depends on the type of growth media used. Previous studies have reported that the MS and Hyponex media are good basal media for protocorm growth of *S. japonica* [27,28]. In the present study, we attempted to find the appropriate MS and Hyponex medium strengths for in vitro growth of *S. japonica*. Our results showed that growth medium type and strength significantly influenced the seedling development of *S. japonica*. Early growth of its seedlings in terms of the fresh weight and leaf area reached a maximum in 1/2 Hyponex medium, which might be because of its high macro- and micro-element contents.

Activated charcoal (AC) has a very fine network of pores, an extremely large surface area, and volume that gives it a unique adsorption capacity [29]. It is often used in plant tissue cultures to improve cell growth and development. In orchid cultivation, charcoal addition has been reported to be related to increased seed germination [30], sprout formation [31], root growth [32], and seedling growth [33]. Our results showed that the addition of AC Hyponex and 1/2 Hyponex media promoted plant growth (Figure 3).

Protocorm and seedling browning often occur during the asymbiotic in vitro seed germination and plant development of several orchid species [34,35,36]. Tissue browning is a severe problem in plant tissue culture. Phenolic browning can be reduced by altering the composition of the growth medium and culture environment, by adding AC, and by adding antioxidants. AC has been used to control explant browning in various plant species [36,37,38]. It can have beneficial and harmful effects on culture media, depending on the medium, explant, and plant growth regulators used. The beneficial effects of AC are frequently attributed to its adsorptive properties. A previous study showed that the addition of AC prevented browning by adsorbing phenolic compounds and inactivating polyphenol oxidase and peroxidase [39]. The well-known positive effect of AC on the promotion of asymbiotic germination and seedling growth in many terrestrial orchids was also confirmed by our results for *S. japonica* in the Hyponex medium with charcoal.

However, seedling browning was observed in plants grown in MS and 1/2 MS media with added AC (Figure 3). Pan and Staden [40] reported that a lower number of roots per explant was obtained after the addition of a high concentration of AC to the growth medium. AC is known to adsorb a number of compounds that are normally incorporated into the culture medium, such as auxins, cytokinins, abscisic acid, vitamins, and iron chelates [41,42,43,44,45]. It is possible that the addition of high concentrations of AC may induce nutrient deficiencies in the culture medium, and such deficiencies can affect plant growth. In our study, the browning of seedlings grown in the MS medium was presumed to be the result of the adsorption of other nutrients by AC. This negative effect of AC was not observed when organic matter was added; this led to an increase in plant growth (Figure 4).

In vitro growth and development of plant tissues can be enhanced by the addition of various organic supplements, such as apple juice, banana homogenate, potato homogenate, coconut water, corn extract, yeast extract, and casein hydrolysate [19,46,47,48]. Apart from them being a natural source of carbon, the reason why organic additives are added into culture media is because they contain natural vitamins, fibers, hormones, and proteins [49]. Banana and apple homogenates may promote seedling formation because they contain nutrients and adequate amounts of sugars which are then supplied to the culture medium. Zeng et al. [50] found that in *Paphiopedilum wardii*, Hyponex medium supplemented with banana homogenate was the most suitable for seedling formation. Arditti [51] reported that BH was added to orchid media to stimulate the differentiation and growth of shoots at later plant development stages. Other researchers observed that the growth of *Renanthera imschootiana* [52] and *Phalaenopsis amboinensis* plantlets [53] increased when BH was added to the growth medium during the culture period. Positive effects of apple juice on orchid seedling growth have also been reported [54]. Our results were similar to previously reported results that showed that supplementation with apple powder along with banana had significant effects on the growth and development of *Phalaenopsis* during protocorm-like bodies (PLBs) development and seedling growth [55]. It can be concluded that banana and apple extracts could be useful ingredients of an organic medium for the in vitro production of *S. japonica* orchids.

Previous studies reported that culture media containing a low level of mineral nutrients showed the best asymbiotic in vitro seed germination of orchids [35,56,57]. Takahsshi et al. [58] performed various conditions to establish the best conditions for *P. radiata* and confirmed that Hyponex medium with organic additives is the best for asymbiotic in vitro seed germination and growth of *P. radiata*. Zahara et al. [59] mentioned that using organic additives can improve the effectiveness of these culture media. In this study, the optimal conditions for asymbiotic in vitro seed germination and growth of *S. japonica* were confirmed, which contained low levels of mineral nutrients and organic additives. These results were identified in culture media conditions of asymbiotic in vitro seed germination and growth of *S. japonica*, and the developed media could be used for the proliferation of endangered species such as *S. japonica*.

## 4. Materials and Methods

### 4.1. Plant Material and Surface Sterilization of Pods

*S. japonica* seeds were collected from Bijarim Forest on Jeju Island (33°29‘18N, 126°48‘33E), Korea in December 2019. Mature pods were washed with Tween20 detergent under running tap water, after which they were immersed in 70% ethyl alcohol for 3 min and surface-sterilized in 20% sodium hypochlorite solution for 15 min. Then, they were washed five times with sterile water, disinfected in 70% EtOH for 3 min, and rinsed with sterile water. The sterilized pods were then dissected longitudinally into two halves using a sterile surgical blade inside a pre-sterilized laminar airflow cabinet. They were then cleaved using a scalpel blade, and the seeds were scraped.

### 4.2. Culture Media for In Vitro Seedling Growth

To evaluate the influence of different strengths of MS (Duchefa, Haarlem, Netherlands) and Hyponex (Kisan Bio, Seoul, Korea) media on seedling growth, the protocorms were grown in four media: (1) MS; (2) half-strength MS (1/2 MS); (3) Hyponex (N:P:K = 7:6:19, 3 g·L^−1^); and (4) half-strength Hyponex (1/2 Hyponex, 1.5 g·L^−1^). To evaluate the effects of activated charcoal (AC; Duchefa, Haarlem, Netherlands) on seedling growth, the protocorms of *S. japonica* were grown in the following four media: (1) MS with AC 0.6 g·L^−1^; (2) 1/2 MS with AC 0.6 g·L^−1^; (3) Hyponex with AC 0.6 g·L^−1^; and (4) 1/2 Hyponex with AC 0.6 g·L^−1^. To evaluate the effects of organic supplements on seedling growth, the protocorms were grown in the following four media supplemented with banana homogenate (Kisan Bio, Seoul, Korea) 30 g·L^−1^ (BH) and apple homogenate (Kisan Bio, Seoul, Korea) 10 g·L^−1^ (AH): (1) MS with BH 30 g·L^−1^ and AH 10 g·L^−1^; (2) 1/2 MS with BH 30 g·L^−1^ and AH 10 g·L^−1^; (3) Hyponex with BH 30 g·L^−1^ and AH 10 g·L^−1^; (4) 1/2 Hyponex with BH 30 g·L^−1^ and AH 10 g·L^−1^. A medium without BH and AH supplementation was used as the control treatment. Subculture was conducted at intervals of about 2 months during the experiment, and after nine months of culture. To evaluate the effects of different media on plantlet development, plant growth was measured in sixteen media; (1) MS; (2) 1/2 MS; (3) Hyponex; (4) 1/2 Hyponex; (5) MS with AC 0.6 g·L^−1^; (6) 1/2 MS with AC 0.6 g·L^−1^; (7) Hyponex with AC 0.6 g·L^−1^; (8) 1/2 Hyponex with AC 0.6 g·L^−1^ (9) MS with BH 30 g·L^−1^ and AH 10 g·L^−1^; (10) 1/2 MS with BH 30 g·L^−1^ and AH 10 g·L^−1^; (11) Hyponex with BH 30 g·L^−1^ and AH 10 g·L^−1^; (12) 1/2 Hyponex with BH 30 g·L^−1^ and AH 10 g·L^−1^; (13) MS with AC 0.6 g·L^−1^, BH 30 g·L^−1^, and AH 10 g·L^−1^; (14) 1/2 MS with AC 0.6 g·L^−1^, BH 30 g·L^−1^, and AH 10 g·L^−1^; (15) Hyponex with AC 0.6 g·L^−1^, BH 30 g·L^−1^, and AH 10 g·L^−1^; (16) 1/2 Hyponex with AC 0.6 g·L^−1^, BH 30 g·L^−1^, and AH 10 g·L^−1^.

All media were supplemented with 30 g·L^−1^ sucrose and solidified with 0.8% (*w/v*) agar. The pH of the media was adjusted to 5.7, and the media were autoclaved at 120 °C for 15 min. Incu Tissue (72 × 72 × 100 mm; SPL, Gyeonggi-do, Korea) filled with 100 mL of media was used as a culture bottle, and all cultures were incubated at 25 ± 3 °C under a photoperiod of 16/8 h light/dark cycle with fluorescent lamps (150 μEs^−1^m^−2^). After seven and nine months of culture, the growth characteristics of the plantlets in different media were compared.

### 4.3. Measurement of Seedling Development and Statistical Analysis

Each treatment consisted of three independent replicates, with five culture bottles per replicate. Seven and nine months after sowing, representative seedlings were chosen and photographed. The fresh weight of seedlings, width of leaves, length of roots, and the number of roots were recorded. Data were subjected to one-way analysis of variance (ANOVA). Differences were considered significant at *p* ≤ 0.05.

## 5. Conclusions

We established a protocol for in vitro propagation of *S. japonica.* The recommended medium for the early and late growth stage of *S. japonica* seedlings differed. In the early stage, seedling growth was enhanced when PLBs were cultured in 1/2 Hyponex media containing BH 30 g·L^−1^ and AH 10 g·L^−1^. However, in the late stage, seedlings exhibited vigorous growth and root development in 1/2 MS medium containing AC 0.6 g·L^−1^, BH 30 g·L^−1^, and AH 10 g·L^−1^. Nutritional requirements for the optimal growth of plant tissues in vitro may vary among species. Therefore, no single medium can be suggested as entirely satisfactory for all types of plant tissues and organs. The protocol proposed in the present study is suitable for germplasm conservation and mass propagation of *S. japonica*, an endangered terrestrial orchid.

## Data Availability

Data is contained within the article.
